# Vicarious Post-traumatic Growth in Professionals Exposed to Traumatogenic Material: A Systematic Literature Review

**DOI:** 10.1177/15248380221082079

**Published:** 2022-04-29

**Authors:** Alexandra Tsirimokou, Juliane A. Kloess, Sonia K. Dhinse

**Affiliations:** 1 Centre for Applied Psychology, School of Psychology, 1724 University of Birmingham, Birmingham, UK

**Keywords:** vicarious trauma, traumatisation, vicarious post-traumatic growth, mental health professionals, professionals’ well-being

## Abstract

Repeated exposure to traumatogenic material as part of work with traumatised individuals can have detrimental effects on professionals’ well-being. Growing research has explored this phenomenon, known as ‘vicarious traumatisation’. Nevertheless, little research has focused on the positive effects of this work on professionals, namely, ‘vicarious post-traumatic growth’. This literature review aims to identify existing research demonstrating mental health professionals’ experiences of growth, along with environmental and personal factors that facilitate this growth. Eight qualitative and seven quantitative articles were identified following a systematic search of six electronic databases and assessed for their quality using standardised checklists. Qualitative studies were assessed using the Quality Appraisal Checklist for Qualitative Studies (NICE, 2012), and quantitative studies were assessed using the Appraisal Tool for Cross-Sectional Studies (Downes et al., 2016). Professionals described changes in the way they view themselves, the value they place on their relationships and their appreciation for life. Important organisational factors and personal traits were identified as significant predictors for professionals’ growth. Our findings have the potential to inform practical recommendations and directions for future research.

## Vicarious Trauma

Professionals working with trauma survivors are repeatedly exposed to highly distressing material as part of their work. Trauma stories can raise responses that disrupt the professional’s internal world and existing beliefs about the self and others (Janoff–Bulman, 1989). The appraisal of clients’ stories can affect professionals’ feelings of a fair and controllable world, which over time can have a major impact on personal and professional well-being (Park & Folkman, 1997).

Research on constructs such as compassion fatigue (CF), secondary traumatic stress (STS) and vicarious traumatisation (VT) has largely developed in the past decades, contributing to an increase of service focus on staff well-being (e.g. National Fund, 2021). These terms are frequently used interchangeably in the literature to refer to the negative effects of routine exposure to distressing material and personal accounts of traumatic experiences. However, several differences across the terms are noted. Compassion fatigue is a phenomenon occurring over time, characterised by emotional and physical exhaustion that hinders the professional’s ability to be empathetic and compassionate (Figley, 1995). Secondary traumatic stress refers to acute physiological and psychological symptoms often resembling those of direct victims’, including increased fear and anxiety, feelings of guilt, sadness or anger, sleep difficulties, intrusive images, as well as physical exhaustion (Figley, 1995). Finally, vicarious trauma refers to the profound shift in world view that occurs in five key areas: trust, safety, control, esteem and intimacy (Pearlman & Saakvitne, 1995). These can be associated with negative changes in terms of a professional’s self-image and view of the world, as well as increased feelings of hopelessness, pessimism and cynicism (McCann & Pearlman, 1990).

As such, it is clear that routine exposure to traumagenic material can be linked to overall decreased well-being, reduced physical and mental health, as well as impaired intimate relationships (Rizkalla & Segal, 2019). Professionals’ compromised well-being in turn is likely to be impeding the quality of therapeutic relationships, service provision and therapeutic outcomes (Delgadillo et al., 2018). Nevertheless, growing evidence suggests that such exposure to traumatogenic material through work with trauma survivors can also have a positive effect. This process is thought to mirror the experience of direct victims of trauma, referred to in psychological literature as stress-related growth (Park et al., 1996), adversarial growth (Joseph & Linley, 2005) and post-traumatic growth (Tedeschi & Calhoun, 1995).

## Post-Traumatic Growth

The relationship between trauma and growth in direct victims of trauma has been explained by different perspectives. For instance, Aldwin and Levenson (2004) argue that the direction of change after a traumatic event depends on the nature of the traumatic event as well as the individual’s resources at the time of the event. Therefore, negative and positive consequences have been characterised as two distinct phenomena. On the other hand, Tedeschi and Calhoun (1995) claimed that both distress and growth from trauma can be experienced simultaneously, or growth can be experienced as a result of the distress. Consistent with this theory, recent studies have indicated that the negative and positive effects of trauma can coexist (Zięba et al., 2019) with post-traumatic growth often developing as an adaptive response to long-term distress (Kjellenberg et al., 2014). Nevertheless, Ramos and Leal (2013) also highlight several factors that may be predictive of post-traumatic growth, such as personality and environmental characteristics, coping, social support and spirituality.

More specifically, research has indicated that growth happens within five broad domains: strength, new possibilities, human relationships, appreciation for life and spirituality (Tedeschi & Calhoun, 1995). As such, survivors of trauma have described experiences of deeper self-discovery, resulting in a strong ‘survivor identity’, and enhanced sense of self (Sheridan & Carr, 2020). Furthermore, narratives of trauma survivors have indicated an increase in their perceived importance of life, feelings of control over one’s self-management, improved personal relationships and spiritual engagement (Slade et al., 2019).

Over the years, as research has increasingly explored positive psychological change after traumatic life events, studies have also started to investigate this phenomenon in individuals vicariously exposed to trauma, such as family members, carers, as well as professionals.

## Vicarious Post-Traumatic Growth in Professionals

The small body of research on vicarious post-traumatic growth (VPTG) has shown that those listening to trauma victims’ narratives can demonstrate significant levels of growth, as can those who experience direct trauma. For instance, Arnold and his colleagues (2005) explored experienced growth in a sample of 21 psychotherapists, who described gains in similar domains outlined above: strength, new possibilities, human relationships, appreciation for life and spirituality (Tedeschi & Calhoun, 1995). Professionals working with trauma survivors may therefore experience personal and professional growth as a result of being witness to their clients’ resilience and ability to overcome adversity, suggesting a vicarious phenomenon that is both positive and very powerful. These experiences may involve changes in self-perception, interpersonal relationships and philosophy of life (Tedeschi & Calhoun, 1995), as well as increased levels of compassion, sensitivity and insight, and appreciation for different spiritual paths and individuals’ own lives (Arnold et al., 2005). In addition, growth unique to the context of one’s professional identity could be noted, whereby professionals experience feelings of job satisfaction and self-competence as a result of witnessing their clients’ growth (Guhan & Liebling-Kalifani, 2011).

To date, few studies have explored VPTG in professionals. A systematic review conducted in 2015 (Manning-Jones et al., 2015) assessed the methods and measures used to assess VPTG, factors that have been implicated to facilitate it, as well as the relationship of VPTG with direct post-traumatic exposure, and STS in professionals. Consequently, while important relational and methodological aspects of the literature are highlighted, certain questions remain unanswered. For instance, to our knowledge, no review has endeavoured to explore growth from an experiential focus, defining what the experience of VPTG is like for professionals. Furthermore, significant inconsistencies were uncovered across studies in terms of predictive factors of VPTG, potentially due to the range of professionals used. Narrowing down the type of professionals used in the studies under review may therefore provide more consistent results. Finally, as research on this topic continues to grow, studies conducted since Manning-Jones et al.’s (2015) review may have yielded more conclusive findings on the predictive power that certain psychological, cognitive, behavioural, interpersonal and external factors play in VPTG.

## The Present Review

This literature review aims to provide an overview of the existing literature in the area of VPTG in the context of mental health professionals, while exploring internal and external factors that may facilitate this phenomenon. Due to the scarcity of research on this topic, both qualitative and quantitative studies were included for review, having the potential to add valuable information from different perspectives. An overview of the most recent evidence base will highlight gaps that continue to exist in the literature, and thereby inform practical recommendations and directions for future research.

From this literature review, it is hoped that professionals and organisations become aware of the process of growth and the factors that contribute to it. Results can have implications both on professionals’ level of insight, as well as organisations’ support strategies for professionals in their work with trauma survivors. This will further safeguard professionals’ and clients’ well-being, reducing staff turnover and sickness and enhancing engagement with and satisfaction from services.

## Method

### Search Strategy

A search strategy was developed to identify articles that were relevant to the research question. Electronic searches were conducted on six databases on 29th January 2021. These included Scopus, Web of Science, PubMed, PsychINFO, EMBASE and Medline. To identify all articles relevant to the research question, a broad range of search terms was utilised. These centred around aspects of (i) post-traumatic growth, (ii) vicarious or secondary exposure to trauma and (iii) a population of professionals. The same search strategy was applied to all six databases. The results from the searches were exported to the referencing manager software EndNote.

### Inclusion and Exclusion Criteria

Specific inclusion and exclusion criteria were applied to articles produced by the search. Articles presenting original data, with a quantitative or qualitative methodology, were included. The phenomenon that was explored was VPTG in professionals who work in a mental health setting. This excluded any studies that looked at professionals’ experiences of CF and secondary traumatisation, as well as those conducted in physical health settings or with non-professionals such as family members. Articles had to have been peer-reviewed, written in English and the full text accessible through available means.

### Search Results

**Figure 1.**
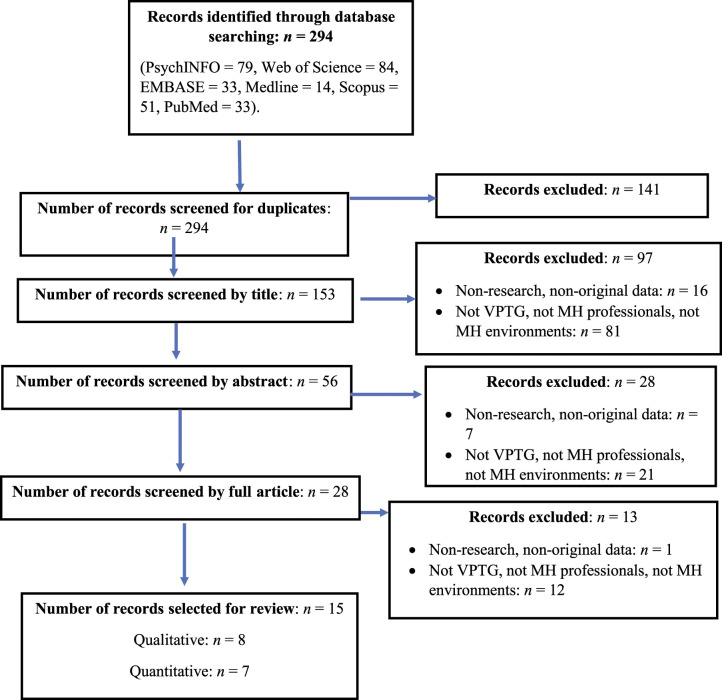
Flow diagram of screening of articles based on identified criteria.

A total of 15 studies, with eight using a qualitative methodology and seven using a quantitative methodology, were identified for inclusion. The search across the six databases resulted in a total of 294 articles. After the removal of duplicates, 153 titles were screened for eligibility. Abstracts of the remaining 56 articles were screened based on the inclusion and exclusion criteria. Following this, 28 articles were reviewed in full, which resulted in the final set of 15 articles being included in the review. Figure 1 shows the PRISMA diagram of the search results and screening procedures followed based on the above criteria.

### Overview of the Studies

Table 1 presents a summary of the 15 articles that were included in the review.

**Table 1.** Overview of study characteristics included in the review.

Study	Aims	Study design	Participants	Measures	Data analysis	Findings
Barrington et al. (2014)	Provide an understanding of VT and VPTG and suggestions on how to effectively manage clinician distress and foster opportunities for personal growth.	Longitudinal Qualitative Design – two interviews, two timepoints apart.	12 clinicians working with people from refugee backgrounds.	Interviews	IPA	Changes in self-perception, changes in life philosophy, increased sense of gratitude, changes in interpersonal relationships, strength from watching people cope, own development of coping strategies, spiritual changes and sense of purpose.
Coleman et al. (2021)	Explore positive and negative impact of working therapeutically in complex psychological trauma. – Focus on positive gains and growth.	Qualitative interviews	21 counsellors, psychotherapists and psychologists from NHS specialist trauma services, CMHT and specialist sexual assault counselling organisation.	Interviews	IPA	Stronger sense of self, finding meaning in their own life and recognising importance of their role.
Hernandez et al. (2015)	Examine vicarious resilience and vicarious trauma in work with torture survivors.	Qualitative interviews	13 US mental health providers working in torture treatment centres in West, East and Midwest.	Semi-structured interviews	Grounded Theory	Changes in self-perception, greater appreciation of own personal circumstances.
Hyatt–Burkhart (2014)	Understanding the experience of mental health workers working with traumatised children and adolescents.	Qualitative interviews	12 US mental health workers in a residential treatment facility for traumatised children and adolescents.	Interviews and focus groups using semi-structured interview.	IPA	Changes in self-perception and philosophy of life, appreciation of life, positive shift in relationships and greater sense of possibilities/hope for human resilience.
Puvimanasinghe et al. (2015)	Explore the experiences of mental health, physical healthcare and settlement workers caring for refugees and asylum seekers in South Australia.	Qualitative interviews	26 service providers for refugees and asylum seekers: doctors, nurses, counsellors, psychologists, managers, project coordinators and case workers.	Semi-structured face to face interviews.	Inductive thematic analysis	Empowerment in witnessing outcomes of work, and work satisfaction.
Ren et al. (2018)	Examine peoples’ lived experience of personal transformation in long-term disaster mental health services.	Qualitative interviews	10 psychological counsellors	Face to face semi-structured interviews.	Inductive thematic analysis	Inner sense of ‘wholeness’, changes in views of life, increased mindful presence and acceptance, empowerment from witnessing resilience and transformation in clients.
			3 psychiatric nurses			
			4 psychiatrists			
			6 social workers			
Silveira and Boyer (2015)	Explore positive effects of trauma work in counsellors of child and youth victims of interpersonal trauma.	Qualitative interviews	4 female counsellors working with children and youth victims of interpersonal trauma.	Individual semi-structured interviews.	Multiple Case Study thematic analysis	Self-development, increased sense of gratitude, changes in personal relationships, changes in views of human strength, increased acceptance and feelings of reward from their work.
Splevins et al. (2010)	Investigate the experiences of interpreters working with trauma survivors – specific focus on VPTG.	Qualitative interviews	8 interpreters working in a therapeutic setting with asylum seekers and refugees.	Face to face semi-structured interviews.	IPA	Changes in self-perception, changes in ‘philosophy’ of life, increased appreciation for life, changes in relationships, empowerment from witness of growth, increased coping strategies and increased acceptance of hardship.
Ben–Porat, 2015	Growth among different populations of therapists – comparison of family violence therapists versus social service department therapists.	Cross-sectional design comparing two groups.	*Group 1:* 143 family violence therapists	Post-Traumatic Growth Inventory, Secondary Traumatic Stress Scale, Sociodemographic Questionnaire, Self-Esteem Scale, Sense of Role Competence, Multidimensional Scale of Perceived Social Support and Colleague Support Questionnaire.	- ANOVAs for differences between groups	- Profession explained variance in growth (SS VPTG higher compared to DV therapists)
						- Secondary traumatisation contributing to VPTG.
					- Hierarchical regression analyses to examine variance explanation of variables.	*Groups separately:*
						- DV therapists: more years of experience in the field linked to lower growth.
			*Group 2:* 71 social service department therapists (total of 214 participants)			- SS therapists: more years of experience in the field linked to higher growth.
Brockhouse et al., 2011	Examine moderators of the relationship between vicarious trauma and growth: sense of coherence, empathy and perceived organisational support.	Cross-sectional design	118 therapists	Demographic information, Jefferson Physician Empathy Scale, Sense of Coherence scale, Perceived Organisational Support Scale and Post-Traumatic Growth Inventory.	- Descriptive statistics	- Strong sense of coherence negatively predicted growth.
						- Empathy was a positive predictor of growth.
			Private practice (27%)		- Single sample t-test	- Organisational support did not predict growth.
			Public sector (32%)			*Other correlations* :
			Combination (41%)		- Regression (moderation analyses)	- Working as part of a clinic linked to higher levels than working in private practice.
Manning-Jones et al., 2016	- Explore relationship between coping strategies and psychological outcomes of vicarious traumatic exposure – moderating power of: social support, self-care and humour.	Cross-sectional design	365 health professionals:	Demographic information, Years of Experience, Secondary Traumatic Stress Scale, Post-Traumatic Growth Inventory, Social Support Scale, Self-Care Utilisation Questionnaire and Humor Styles Questionnaire (Self-Enhancing Humor subscale).	- Descriptive statistics	- Humour, self-care and peer support positively predicting VPTG.
			44 doctors			
			76 nurses		- T test -gender differences	- Social workers experiencing highest level of STS and VPTG.
	- Differences between five groups of professionals.		103 social workers		- Pearson’s correlations - coping	
			70 psychologists		- Stepwise regression variance predictors on outcome.	- Psychologists experiencing the lowest for both.
			72 counsellors		- MANOVA -differences by profession	
O’Sullivan & Whelan, 2011	Investigate level of VPTG among telephone counsellors – influence of different psychological and environmental factors.	Cross-sectional design	64 telephone counsellors from 5 different organisations.	Demographic questionnaire, Post-Traumatic Growth Inventory, Professional Quality of Life Scale, Jefferson Scale of Physician Empathy and Crisis Support Scale.	- Descriptive statistics	- Compassion fatigue significantly related to PTG.
					- Pearson’s correlations – relationships between variables.	- The more calls taken per shift, the less growth.
					- Multiple regression – predictors on VPTG.	- Empathy and crisis support were not significant predictors of growth.
Rhee et al., 2013	Measuring level of VT, VPTG and other factors that affect VPTG in child protective service workers.	Cross-sectional design	204 child protection social workers from 43 child protective agencies.	Demographic information, Post-Traumatic Growth Inventory, Impact of Event Scale, Questions measuring social support ‘How much support have agencies provided in crises? Have you sought support from them? Have you obtained counselling from them?’ + level of satisfaction with colleagues and supervisor relation, Crisis Support Scale and Coping Strategy Indicator.	- Descriptive statistics	- Pursuing social support as a way of coping with stress was the strongest predictive factors of VPTG.
						- Higher scores with regard to invasion meant higher VPTG.
					- T-test	- Higher scores of growth for religious workers.
			Workers working with child abuse.		- One-way ANOVA	- Higher scores of growth for those who maintained friendly relationships with colleagues and supervisors.
					- Regression analysis	- Receipt of independent counselling was helpful.
Rizkalla and Segal, 2020	Examine work stressors and organisational support and their associations with VPTG and intimate relationships.	Cross-sectional design	317 aid workers from organisations in Jordan.	Sociodemographic information, Trauma work stressors (created for the study), Trauma and Attachment Beliefs Scale, Organisation support (created for the study), The Needs at Work Assessment (created for the study), Finding meaning, Differentiation of Self Inventory-Revised, Post-Traumatic Growth Inventory and Personal Assessment of Intimacy in Relationships.	- Univariate descriptive statistics	- Increased VT associated with increased VPTG, decreased intimacy and decreased differentiation.
					- Correlations	- Increased needs addressed by NGOs were associated with increased VPTG, differentiation and finding meaning. VPTG not linked to increased co-worker support.
					- Bootstrap analyses to examine the specific direct path estimates between variables.	- Higher differentiation associated with decreased VPTG, and increased intimacy.
					- Missing data	- Increased finding meaning associated with increased VPTG and intimacy.
Zerach et al., 2015	- Examine PTSD, ST and VPTG among psychiatric nurses compared to community nurses.	Cross-sectional design	196 Israeli nurses:	Sociodemographic characteristics, PTSD inventory, Post-Traumatic Growth Inventory, Professional Quality of Life Scale, Multi-dimensional Health Locus of Control Scale, Potential vulnerability statements (created for the study), Life Events Checklist and Exposure to Stress Questionnaire.	- Descriptive statistics	- Psychiatric nurses higher levels of PTSD and ST symptoms, but lower levels of VPTG.
					- Pearson’s correlations effects of sociodemographic variables with outcome variables.	
	- Contributions of providing an aetiology (sense making/meaning giving) symptoms.		90 psychiatric nurses		- ANOVA, MANOVA	- ST symptoms positively related to VPTG among CN, but negatively associated among PN.
					- T-tests	
	- Community clinic nurses used as a comparison group.		106 community clinic nurses		- Hierarchical regression	- Exposure to patients’ violence, PTSD and ST symptoms, along with illness attribution dimensions of ‘powerful others’, predicted nurses’ VPTG.

*Note.* VT = vicarious traumatisation; VPTG = vicarious post-traumatic growth; DV = domestic violence.

#### Methodology of the Studies

Eight of the studies adopted a qualitative methodological approach and seven of the studies adopted a quantitative methodological approach. One of the qualitative studies was of a longitudinal nature, conducting two interviews at two different timepoints a year apart (Barrington & Shakespeare–Finch, 2014). The other seven qualitative studies conducted semi-structured interviews, with the exception of one study (Hyatt–Burkhart, 2014), which undertook an additional focus group. The majority of the qualitative studies (*n* = 4) used Interpretative Phenomenological Analysis to analyse the data (Barrington & Shakespeare–Finch, 2014; Coleman et al., 2021; Hyatt-Burkhart, 2014; Splevins et al., 2010). The remaining qualitative studies utilised a Grounded Theory approach (Hernandez-Wolfe et al., 2015), an Inductive Thematic Analysis approach (Puvimanasinghe et al., 2015; Ren et al., 2018) and a Multiple Case Study approach (Silveira & Boyer, 2015).

The seven quantitative studies were cross-sectional in design, looking at the data in a chosen population at one specific timepoint. Comparisons between different groups of professionals were performed for four quantitative studies (Ben-Porat, 2015; Brockhouse et al., 2011; Manning-Jones et al., 2016; Zerach & Shalev, 2015). All seven quantitative studies gathered sociodemographic information and administered the Post-Traumatic Growth Inventory measure (Tedeschi & Calhoun, 1996). Studies used various other measures to explore factors in relation to VPTG: STS (*n* = 2) and PTSD (*n* = 1), Self-Esteem (*n* = 1), Sense of Role Competence (*n* = 1) and Sense of Coherence (*n* = 1), Perceived Social Support (*n* = 1) and Organisational/Colleague Support (*n* = 2), Empathy (*n* = 2), Self-Care Utilisation (*n* =1) and Coping (*n* = 1), Humour (*n* = 1), Relationship Intimacy (*n* = 1), Professional Quality of Life (*n* = 2) and Crisis Support (*n* = 2), Trauma Beliefs (*n* = 1) and Locus of Control (*n* = 1). Data were predominantly analysed using stepwise, multiple or hierarchical regression analyses. Differences between groups were explored using t-tests and ANOVAs.

#### Participant Samples

A total of 1597 participants were included across the 15 studies. Of these, in 14 studies, 411 were male and 1129 were female. One study did not report participant gender. The age of the participants ranged from 18 to 73 (*M* = 43.5, *SD =* 7.3) across the 11 studies that reported age. Participants were mental health professionals working in the private or public sector of general mental health services (*n* = 3), as well as in various specialist services, such as Domestic Violence (DV) centres (*n* = 1), Refugee and Asylum Seeker support services (*n* = 4), Complex Psychological Trauma services (*n* = 1), Torture Treatment Centres (*n* = 1), Telephone Counselling centres (*n* = 1), Natural Disaster Support Services (*n* = 1), Youth Trauma services (*n* = 2) and Child Protection services (*n* = 1). The years of experience of professionals ranged from 1 to 50 years (*M* = 10.4, *SD =* 6.2), based on data derived from 10 studies that provided this information.

The studies were conducted in various different countries, such as the United Kingdom (*n* = 3), Australia (*n* = 3), Israel (*n* = 2), the United States (*n* = 2), New Zealand (*n* = 1), China (*n* = 1), Korea (*n* = 1), Jordan (*n* = 1) and Canada (*n* = 1). As such, multiple nationalities of participants were reported, including South American, African, Australian, Syrian, Israeli, North American, Jordanian, New Zealander (Pakeha and Maori), Chinese, Korean, Canadian, French, Iraqi, Iranian and British. The sample across studies could therefore be considered ethnically diverse, and representing both Western and non-Western countries.

### Quality Assessment

All articles were individually assessed for quality. It is important to note that all quality ratings were based on the assessment of the first author, and it is possible that others may have rated each item differently. Two articles, one qualitative and one quantitative (13%), were rated by a colleague for inter-rater reliability. Inconsistencies in ratings were reviewed and one change (Coleman et al., 2021) was made in the ratings. This involved the extent to which the context was adequately described in one of the qualitative articles, which was changed from ‘Partial’ to ‘Yes’.

#### Qualitative Studies

The checklist used to assess the quality of the eight qualitative articles was the Quality Appraisal Checklist for Qualitative Studies developed by the National Institute for Health and Care Excellence (NICE, 2012). A few modifications were made to this checklist. The original checklist had one large item called ‘Conclusion’, which was broken into two sub-questions for the purpose of this review. The two questions covered the extent to which findings were clearly grounded in previous literature, and whether strengths and limitations of the study were identified. Furthermore, one final question was added about the disclosure of funding sources and conflicts of interest, as the presence of these could potentially affect the authors’ interpretation of transcripts and theme extraction. Therefore, this is deemed to be an important consideration in the context of qualitative research (Levitt et al., 2017). Each item in the checklist was marked green if the criterion was met, yellow if it was partially met and red if it was not met or reported. An overall rating was then given (see the Supplementary Material for an overview of the quality ratings).

Three qualitative studies met all or most criteria of the checklist and were scored of high quality, four studies met some of the checklist criteria with some aspects that were not adequately described and one study was thought to have not fulfilled the checklist quality criteria to a satisfactory level. The qualitative approach was deemed appropriate for all studies, as they aimed to explore the participants’ lived experiences. The designs chosen were in line with the identified aims, and most studies included an explicit rationale for this. Aims were derived from existing literature and identified gaps.

Six of the eight studies provided clear descriptions of the data collection methods, while two lacked in clarity of how participants were identified, and how their data were stored. Adequate researcher reflexivity was achieved for half of the studies, which provided a clear description of the role of the researcher, and how bias was minimised. Characteristics of the participants and settings were well described, and the overall methodology of the studies was deemed reliable for the majority of them (6/8). For the data analysis, most studies provided a clear account of this process, with two studies remaining relatively vague in their description. Some studies further failed to report the number of researchers involved and how trustworthiness of the analysis was achieved. Results were overall well presented, with direct quotes from participants and in-depth interpretations. These were deemed relevant to the identified aims of the studies and contextualised within the wider literature. The strengths and limitations were considered to a satisfactory level by only three of the eight articles. Four studies provided a clear account of ethics, and seven of the studies acknowledged funding sources and potential conflicts of interest.

#### Quantitative Studies

The checklist used to assess the quality of the seven quantitative articles was the Appraisal Tool for Cross-Sectional Studies (Downes et al., 2016). Each item in the checklist was marked with a ‘Yes’ if the criterion was met (green), ‘No’ if it was not met (red) and ‘Partial’ if it was partially met (yellow). The ‘Partial’ option was added for the purpose of this review, to account for the items whereby neither a ‘No’ nor a ‘Yes’ applied. This tool further allows for items to be marked as ‘Unknown’ if something is not reported (see the Supplementary Material for an overview of the quality ratings).

The quantitative articles overall adopted an appropriate design for their research, looking at the presence of the phenomenon of VPTG at a specific moment in time. In some studies, different groups were compared, allowing for differences between professionals to be highlighted. The sample size was justified for most of the studies that reached a large number of participants, most of which were deemed to be appropriate in representing the target population. Clear inclusion and exclusion criteria for participants were outlined for six of the studies, while the other kept its criteria relatively broad. Sampling was purposive for most of the studies, which could make them open to selection bias and error. Furthermore, online means of reaching participants in their workplace could lead to further influences on recruitment, such as the ‘healthy worker effect’ and self-selection bias. Response rates were not adequately reported, and therefore it is unknown whether these could indicate a non-response bias. Some studies did not provide a clear description of how the number of non-responders was calculated. The majority of the studies further failed to categorise non-responders, although the nature of the design may have not allowed for this.

Predictor and outcome variables for all studies were deemed appropriate and had been trialled and published previously. One study had a large number of measures that had been specifically developed for the study and therefore had not been previously validated. Three out of the seven studies were deemed to have described their methods to an extent that enabled replication. Vague descriptions of some procedures were noted for the remaining studies. On the other hand, Results sections of the studies were of a high standard. All basic data were adequately reported, and clarity around statistical significance was maintained throughout. Statistical results were presented for all measures described, both in the text as well as in tables. All discussion points were justified by the results and grounded in previous literature. Limitations were also considered for all studies. Finally, ethical approval and consent was addressed by six of the seven studies, while the majority (5 out of 7) failed to disclose any funding sources or potential conflicts of interest that could have affected the interpretation of the results.

### Data Analysis

All relevant outcomes were extracted to identify reciprocal and refutational relationships across studies, in line with relevant steps undertaken as part of the method of thematic analysis (Braun & Clarke, 2006). For the qualitative articles, data extraction was performed in a systematic manner. Each article’s identified themes that were relevant to the research question were placed on a data extraction grid, along with a short description and participant quotes. When moving through the studies, a dynamic process took place, whereby similarities, common concepts and characteristics were identified. Each theme was subsequently placed in the corresponding column of perceived similar themes across the studies. Additional columns were created for each new theme. Before these themes were finalised, the extraction grid with the descriptions and participant quotes was revisited to ensure that themes were grouped coherently. A name was given for each cluster of similar themes. Seven main themes and two sub-themes were identified across studies relating to VPTG in professionals. For the quantitative articles, data analysis was performed in a similar way. Outcomes that were generated through the same or a similar measure were grouped to identify reciprocity or refutation across studies in a data extraction grid.

## Results

Results from the qualitative and quantitative studies will be presented separately. This is because the focus of the qualitative studies seemed to be on what growth looks like in professionals, while quantitative articles explored variables that contributed to growth.

### Qualitative Results

A total of seven themes were identified to best represent the findings of the qualitative articles included in this review, some of which had further sub-themes.

#### Self-Development

The first theme of ‘Self-Development’ was found in seven of the eight studies. This was a change in participants’ perceptions of themselves, both at a personal and professional level. Participants realised the strength required to do this work, feeling stronger in themselves and better able to deal with frustration and hardship in their daily life: ‘[The work] makes you strong, I’m a stronger person than I used to be’ (Barrington & Shakespeare-Finch, 2014, p. 1692). Professionals further felt as though they had become more open-minded and tolerant through this work, as well as less judgmental and more flexible with individuals in their lives: ‘[Work has] deepened my understanding of people, and maybe [helped in] being even less judgmental’. (p. 1692). They described themselves as being more adaptable and less likely to be perturbed by the ‘small stuff’, having a reduced tendency to get ‘worked up’ (Hyatt–Burkhart, 2014). Furthermore, participants reported feeling more confident with their professional skills: ‘I think I have developed on different levels but the simplest level it would be having more time and experience, … I have developed skills to help me assist people’ (Coleman et al., 2021, p. 2803), as well as in their personal lives: ‘[I] have more confidence that I can make choices to be involved with people in my life, whether it’s difficult clients or perpetrators or difficult things in my personal life’ (Hernandez–Wolfe et al., 2015, p. 162).

Finally, professionals felt a deeper connection with their existence and their core selves, such as a feeling of ‘wholeness’ (Ren et al., 2018): ‘I would say that I have become a much deeper person, I think deeper. I feel deeper’ (Splevins et al., 2010, p. 1711). Changes in spiritual experiences were also described in two directions. Firstly, participants felt a deepened understanding and connection with their religion: ‘I now feel the power of religion’ (Ren et al., 2018, p. 2320). However, participants also re-considered their faith and re-evaluated their relationship with it: ‘I cannot believe in a God that allows so many people to live through so much suffering’ (p. 1692), ‘I do believe in God and Jesus, but not the way that I used to’ (Barrington & Shakespeare–Finch, 2014, p. 1692). Regardless of the direction of change, all participants experienced this as personal growth. Finally, a few participants also reported becoming less inclined to tolerate acts of injustice, in light of their clients’ experiences: ‘I have a feeling of not wanting to leave things unchallenged when you know there are abuses, however small really’ (Splevins et al., 2010, p. 1711).

#### Changes in Philosophy of Life

Seven of the eight studies contributed to this theme. Participants described changes in their philosophy of life: ‘Your idea about what the world is, like it just kind of develops, and it kind of crumbles, and it grows and blooms in all [of] these weird ways’ (Barrington & Shakespeare–Finch, 2014, p. 1962). They spoke about comparing their life and those of their clients: ‘Looking at life through a lens coloured by a new awareness of what the world is like’, which in turn facilitates this change in philosophy: ‘My life is easy. I have a good life. I have everything I need, everything I want’ (Hyatt-Burkhard, 2014, p. 456). Professionals further identified changes in their core values and priorities: ‘This experience enriched my life, to make me know what is the most important, my family, myself…’ (Ren et al., 2018, p. 2317), ‘…material things [have] lost its importance to me’ (Splevins et al., 2010, p. 1711).

##### Sense of Gratitude and Appreciation

Seven of the eight studies further described a change in life philosophy through an increased sense of gratitude. Professionals spoke about greater appreciation of their own life circumstances as a result of hearing their clients’ stories: ‘I appreciate my life a lot more after sitting with people that [have] just [had] the most basic rights taken away, and the most basic needs [taken away]’ (p. 1692), ‘Knowing that these people do not even have one percent of what we have in our lives, helps you to appreciate what you have and to be grateful, and not to whine about things’ (Barrington & Shakespeare-Finch, 2014, p. 1692). Participants noticed an added value of smaller things in their life, whereby ‘simple’ things such as ‘warmth, plenty to eat, safety and security’ are faced with a deeper appreciation (Hyatt-Burkhart, 2014). Hearing other people’s challenges and witnessing how these are faced helped professionals put their own challenges into perspective and acknowledge their strengths (Silveira & Boyer, 2015). Increases in experiences of ‘being present’, ‘living in the moment’ and ‘being mindful’ were also described as a result of hearing clients’ difficult life stories: ‘I feel life is uncontrolled, you never know what you will face in the next moment. All I can do is to treat every second seriously, to focus on every moment…’ (Ren et al., 2018, p. 2318).

##### Acceptance

Participants in two studies described greater willingness for ‘acceptance’ about the existence of imperfections, both in life as well as in themselves (Silveira & Boyer, 2015). Participants perceived greater flexibility and ‘softened’ reactions to negative life experiences, as well as growth towards being unbothered by uncertainty, unpredictability and challenges: ‘I treat adversity as a normal part of life, because life will move on in its own way’ (Ren et al., 2018, p. 2318).

#### Change in Personal Relationships

Possibly linked to the theme of a changed life philosophy, professionals further identified changes in their personal relationships. This theme was supported by four of the eight qualitative articles. Professionals noticed changes in the nature of their interactions, while also reducing their social circles to the individuals that they felt most connected to: ‘I think the people that I spend time with has shrunk actually. I think I spend more time with people that I’m closest to and, you know, have really meaningful relationships with, and very little time with other people that I used to see just for socializing’ (Barrington & Shakespeare-Finch, 2014, p. 1692). Furthermore, professionals noticed that hearing stories about unhealthy and ruptured relationships helped them recognise the presence of loving and fulfilling relationships in their lives, while also spurring them on to pay more attention to those (Hyatt-Burkhart, 2014). Participants reported feeling able to apply what they taught their clients during treatment, such as becoming more accepting and compassionate towards people’s imperfections (Silveira & Boyer, 2015), and being more open and intimate with their loved ones (Splevins et al., 2010).

#### Possibilities of Human Resilience

Six of the eight articles identified strong emotional reactions from witnessing the possibilities of human resilience: ‘How can that happen to someone? How can you survive? How can you, like how can you cope and keep living? [...] You see the sadness, but the person keeps going, and you wonder how’ (Barrington & Shakespeare–Finch, 2014, p. 1690). This develops a sense of increased possibilities for human resilience and change, which in turn enhances professionals’ own ability to explore new possibilities and expect positive outcomes. This leads to a growth journey that is reciprocal for the client and the professional: ‘When I saw the recovery from the overwhelming pains, I know there is nothing we cannot pass, they can do it, and I can do it…’ (Ren et al., 2018, p. 2320). Professionals felt inspired by their clients’ ability to access joy despite their lived experiences: ‘That strength and that core…was pretty amazing to be able to witness their experience’ (Silveira & Boyer, 2015, p. 520). From this, professionals realised the human ability to not only survive but also ‘flourish’ after trauma: ‘It was amazing. I was flabbergasted. How he actually turned from how he was to a normal, young, and happy, sort of very positive, child. What I saw in the beginning—what I’m seeing today—was amazing. Amazing. Absolutely fantastic’ (Splevins et al., 2010, p. 1710). This left them hopeful in the knowledge that humans are more durable than they had imagined: ‘Sometimes you’re just surprised by the resilience of some clients; that they are still able to laugh and joke about things, even [though] they went through terrible times in their life. That is incredible!’ (Barrington & Shakespeare–Finch, 2014, p. 1693).

#### Development of Coping Strategies

Professionals in three studies recognised the importance of developing strong coping strategies. They reflected on their strategies at an organisational level, whereby talking to colleagues and utilising supervision were identified as helpful: ‘You won’t keep something inside you and take it home, at least someone is there that can listen to help’(p. 1693); ‘There are [colleagues] around who are able to listen and understand, and that’s great. Without that, I think I wouldn’t be able to work, it would be very hard to work here’ (Barrington & Shakespeare–Finch, 2014, p. 1694). Furthermore, maintaining a work-life balance was vital to facilitate growth through ‘taking a step back’. Numerous self-care strategies were identified with the most common being mindfulness, exercise, connection with nature and reaching out to friends and families. Participants also reported making a conscious effort to find positives in their work: ‘You have to look for the positives, only looking for more negatives is not good’ (Barrington & Shakespeare–Finch, 2014, p. 1694). Finally, participants linked the ‘passage of time’ and customisation to the work environment to an increased ability to cope: ‘With more experience and more training it’s something that I can manage quite well now I’d say’ (Splevins et al., 2010, p. 1711).

#### Work Satisfaction and Sense of Purpose

Participants from four studies identified a great level of satisfaction and sense of purpose as a result of their work. Recognition of their contribution to the well-being of others is seen as part of their growth, as it has armed them with increased confidence in their abilities (Barrington & Shakespeare–Finch, 2014). Professionals felt pride about facilitating growth in their clients and converting their ‘chaos’ into ‘meaning’ (Coleman et al., 2021). Observing clients gradually become more empowered and independent in turn empowers the professional to feel confident about the services they provide (Puvimanasinghe et al., 2015). This is thought to create a strong professional identity and increases passion and motivation for their work (Silveira & Boyer, 2015).

#### Distress into Growth

The final theme identified by two of the studies was the notion that suffering needs to occur in order for growth to follow. Negative emotional responses to traumatised clients are suggested to signify healthy human processes that slowly dissipate and allow positive emotions to dominate: ‘Feeling the distress does help you understand, and without it I don’t know whether you could feel the benefits in the same way, actually. If you experience the distress, you know, even deeper, maybe you feel even more the benefits’ (Splevins et al., 2010, p. 1710). Transformation from suffering to growth is described as a dynamic process, during which existential learning plays a pivotal role in achieving ‘existential integrity’. As such, experiences like burnout, feelings of emptiness, self-doubt, secondary traumatisation and a sense of uncontrollability, come first and are a pivotal step towards a positive transformation (Ren et al., 2018).

### Quantitative Results

The quantitative results highlighted variables that may predict VPTG in professionals.

#### Relationship Between Trauma and Growth

The relationship between vicarious trauma and growth was explored in six of the seven articles. As identified in the qualitative studies, quantitative studies also suggested that higher levels of vicarious traumatic exposure were linked to higher levels of VPTG (Manning-Jones et al., 2016; O’Sullivan & Whelan, 2011; Rhee et al., 2013; *Rizkalla & Segal, 2020). Nevertheless, one study found that rates of VT indeed predicted growth, however, that the relationship was curvilinear (Ben-Porat, 2015). In other words, when secondary traumatisation reaches levels that are too high, opportunities for growth decline. Two further studies supported this idea. Inpatient psychiatric nurses reported higher levels of PTSD and Secondary Trauma in their work compared to community nurses; however, their growth was significantly lower (Zerach & Shalev, 2015). Furthermore, a higher number of calls answered by telephone counsellors predicted reduced growth (O’Sullivan & Whelan, 2011).

Further variables were found to moderate this relationship. For instance, opposite directions of growth were noted for therapists working with DV victims, compared to therapists in Social Services (SS), relating to their years of experience. More experience was associated with lower sense of growth for DV therapists, whereas less experience was related to higher sense of growth for SS therapists (Ben-Porat, 2015). This was thought to be due to the nature of their work, whereby the SS therapists see a broader range of cases, which may allow for higher levels of growth. On the other hand, DV therapists’ work is limited to a specific type of trauma, leading to higher levels of fatigue, possibly impeding growth.

#### Organisational Characteristics

Organisational characteristics were explored in five of the seven quantitative studies. Brockhouse et al. (2011) found that perceived organisational support did not directly predict growth. Nevertheless, comparisons between groups in the same study showed that working as part of a larger clinic was linked to higher levels of growth compared to those in private practice, possibly linked to receiving more informal supervision and higher levels of support from colleagues. Rhee et al. (2013) also demonstrated that higher levels of growth were associated with better relationships with colleagues and supervisors, thought to be linked to the ability to express emotional responses following sessions with clients, while also a level of normalisation of distress demonstrated from colleagues and access to well-being resources in the workplace (Manning-Jones et al., 2016). *Rizkalla & Segal, 2020 demonstrated the importance of perceived needs addressed by organisations in facilitating growth, including the provision of enough resources, benefits, supervision and training on offer for employees. Nevertheless, perceived co-worker support was not a predictor of growth for the same population. Finally, crisis support provision from the organisation was not a significant predictor of growth for telephone counsellors (O’Sullivan & Whelan, 2011).

#### Personal Characteristics

Various personal characteristics were also linked to increased growth in professionals. Firstly, older age of professionals was related to higher VPTG (Brockhouse et al., 2011). Furthermore, past trauma in personal life (Ben-Porat, 2015), as well as receipt of independent counselling (Rhee et al., 2013) were found to be variables that facilitated growth. Additionally, professionals’ humour and level of self-care (Manning-Jones et al., 2016), as well as stronger religious beliefs (Rhee et al., 2013), positively predicted VPTG. Level of empathy was also found to be a positive predictor of growth in therapists (Brockhouse et al., 2011), though O’Sullivan and Whelan, 2011 did not find a significant association between this trait and level of growth in telephone counsellors. Interestingly, a strong sense of ‘understanding’ of the world negatively predicted growth in therapists, which is thought to be linked to less flexibility in accommodating new information about the world, and therefore less opportunity for growth (Brockhouse et al., 2011). Similarly, increased efforts to “find meaning” in the distress was associated with higher levels of VPTG in aid workers (*Rizkalla & Segal, 2020).

Additionally, actively pursuing social support as a way of coping with stress was the strongest predictive factor of VPTG in social workers who worked with child abuse (Rhee et al., 2013). However, Ben–Porat (2015) found non-significant associations between VPTG and support systems in professionals’ lives. This may demonstrate that social support systems are not by themselves significant predictors, but that the individual’s level of active pursuit of support systems, or active use of coping strategies in general, plays a role.

## Discussion

This literature review aimed to provide an insight into the existing evidence base of what VPTG looks like in professionals, as well as internal and external factors that may contribute to and facilitate this phenomenon.

### Summary of Findings

Studies supported the idea that adversity is necessary for growth, as levels of VT were predictors of VPTG. This is in line with existing literature on post-traumatic theory, which suggests that positive accommodation is a trajectory of which traumatisation is an integral part (Joseph & Linley, 2005). Directly or vicariously traumatised individuals go through a process that requires them to reflect on the trauma experience and create new meanings, rebuilding a collapsed view of the world, others and oneself (Zeligman et al., 2019). This process is experienced as growth.

Nevertheless, studies of this review also suggested a curvilinear relationship between trauma and growth, whereby higher exposure to vicarious trauma is linked to lower levels of growth (e.g. O’Sullivan & Whelan, 2011; Zerach & Shalev, 2015). Professionals, such as telephone counsellors and inpatient psychiatric nurses, demonstrated lower levels of growth over time, thought to be linked to higher and continuous exposure to trauma, as well as significantly less opportunities to walk away from it and take a break to reflect. This is in line with research involving direct trauma survivors, suggesting that ongoing continuous trauma is negatively related to post-traumatic growth (Kira et al., 2013). Furthermore, a recent study specifically exploring this relationship found that moderate levels of vicarious trauma are most associated with the highest levels of VPTG in professionals (Dar & Iqbal, 2020).

Similarly, the relationship between years of experience and growth was positive for those with more diverse work, compared to specialist professionals, who showed increased growth at earlier stages in their career, which declined over time. These findings demonstrate that more diverse work could continuously provide new learning opportunities that in turn promote growth and development. On the other hand, highly specialist professionals’ continuous exposure to a specific type of trauma may increase fatigue and burnout over time, and thereby hinder the level of growth.

#### What Does Growth Look Like?

The literature has recognised growth from trauma in three domains, including changes in self-perception, life philosophy and interpersonal relationships (Tedeschi & Calhoun, 1995). This is in line with the changes identified by professionals in the qualitative studies of this review. Changes in ‘self-perception’ involved participants’ professional and personal identities, with reported increases in confidence, resilience and pride. Existing research has demonstrated that professionals who feel higher levels of empowerment in their work environment experience lower levels of STS from their work (Choi, 2017). As such, feelings of empowerment from witnessing clients’ growth are likely to contribute to a positive transformation and an increased sense of purpose, counteracting the negative impact of the work.

Most studies also reported positive shifts in professionals’ life philosophy in the form of greater appreciation of their own personal circumstances (Hernandez-Wolfe et al., 2015), and the smaller things that once went unnoticed (Hyatt-Burkhart, 2014). In line with Joseph and Linley’s (2005) theory of growth, these changes may be linked to an accommodation of an updated self-view of human’s vulnerability and lack of immunity from disaster. This may have given rise to a desire to appreciate life and live it to the fullest. In line with this, studies also demonstrated how professionals’ increased adaptability and resilience enabled them to tolerate life’s challenges and face adversity (Hyatt-Burkhard, 2014; Ren et al., 2018). This can be explained by the term ‘vicarious resilience’, identified as one of the positive effects of working with trauma survivors (Hernandez-Wolfe, 2018).

Finally, professionals reported putting more attention and value on their interpersonal relationships (Hyatt-Burkhart, 2014). Trauma workers described being more open and intimate, and more respectful and less judgmental towards others as a result of their work (Splevins et al., 2010). This is in line with existing literature showing that counsellors working with individuals who struggle with interpersonal relationships have shown increased skills in anger management, assertive and constructive communication skills, as well as higher levels of awareness of their closed ones’ needs (Ben-Porat & Itzhaky, 2015). As such, professionals demonstrating growth in the domain of ‘interpersonal relationships’ may actively apply skills that they are modelling or teaching to their clients in their own relationships, ultimately enhancing their quality (Silveira & Boyer, 2015).

#### What Factors Facilitate Growth?

Various factors that facilitate growth were highlighted. Firstly, professionals’ support system was found to be a significant predictor of growth in numerous studies. General organisational support (*Rizkalla & Segal, 2020), as well as positive relationships with colleagues and supervisors (Rhee et al., 2013), were found to be crucial in mitigating the negative impact of working with trauma. Supportive work peers may enable the expression of reactions to traumatogenic material, while also normalising the distress linked to that (Catherall, 1995). Furthermore, supervision can enhance a professional’s ability to process the traumatic content of their work in a safe space, while also offering helpful perspectives (Knight, 2018). These findings reinforce the importance of services providing professionals with adequate resources and trauma-informed supervision, as well as an open and nurturing environment for potential growth. Professionals’ support system and use of self-care strategies were also linked to significant VPTG (Manning-Jones et al., 2016; Rhee et al., 2013). A recent meta-synthesis of qualitative studies examining vicarious trauma and VPTG revealed that actively choosing to utilise organisational and personal coping strategies help to reduce levels of distress among trauma workers (Cohen & Collens, 2013). This highlights the importance for organisations to be promoting healthy coping strategies, self-care and work-life balance, with an aim to maximise professionals’ overall well-being.

In terms of personal characteristics of professionals, their level of empathy had a positive relationship with growth (Brockhouse et al., 2011; Coleman et al., 2021), as this allows for a deeper connection with the client’s distress, and therefore can have a higher personal impact. In turn, this encourages the development of strategies to accommodate the distress, leading to significant growth. Similarly, professionals who demonstrated higher identification with their clients and lower separation from their work, experienced higher levels of vicarious trauma, but also higher levels of growth (Manning-Jones et al., 2016; *Rizkalla & Segal, 2020). This could suggest that empathy is not only beneficial for the therapeutic relationship and the client (Feller & Cottone, 2003), but also for the therapist’s healing and growth from vicarious trauma. In fact, research has demonstrated that a stronger therapeutic connection predicts mutual growth in both the client and the therapist (Engstrom et al., 2008).

A higher sense of coherence in professionals was a negative predictor of VPTG (Brockhouse et al., 2011). Sense of coherence represents the extent to which the professional views the world as comprehensible and manageable (Super et al., 2015). A more coherent therapist may therefore have less opportunity to go through the process of positively accommodating new information, and therefore have less opportunity for growth. In relation to this, higher attempts to ‘find meaning’ in the trauma and humour were associated with increased VPTG (Manning-Jones et al., 2016; *Rizkalla & Segal, 2020). These are suggested to be linked to a more positive and flexible approach to life in the face of adversity, and an openness to finding a new perspective on initially distressing situations (Moran & Shakespeare-Finch, 2003). In turn, flexible approaches when faced with adversity have been linked to increased resilience and active use of available resources (Masten, 2007).

### Implications of Findings

The integrative model of stress and coping by Schaefer and Moos (1998) suggests a set of determinants for positive changes following traumatic events. According to this model, positive change can be predicted by the following factors: the pre-trauma resources, such as individual traits and surrounding support, the characteristics of the trauma, such as the severity and length of the exposure, and post-trauma factors, such as coping strategies and surrounding support. This highlights the importance of organisations committing to their staff’s well-being by providing adequate resources for support. These resources include the provision of specialist training on vicarious trauma and practices of self-care for all professionals, the availability of regular peer and individual trauma-informed supervision, and promotion of self-reflection on the work’s personal impact on each professional.

It is vital that professionals are educated to identify the signs of vicarious trauma, while also having various opportunities to reflect on this within a safe space, as well as becoming aware of their potential for growth through this work, and being consistently encouraged to actively reflect on their positive experiences. Adopting a growth-based mindset is likely to be empowering for professionals, increasing their well-being and resilience, and contributing to employee satisfaction and staff retention. Various elements of growth can be incorporated into training, peer or individual supervision and professional conferences. In turn, this may mitigate the relationship between one’s work and vicarious trauma.

Furthermore, based on the above results, close attention needs to be paid to staff in specialist services who are exposed to the same type of traumagenic material every day, as well as those who deal with large volumes of clients. Professionals should be supported to take regular breaks between their clients and use this time to process this material. Not exceeding the hours of work and ensuring a work-life balance is vital for professionals to avoid the impact of ongoing vicarious exposure to trauma and promote growth (Ireland & Huxley, 2018). These are important characteristics that all organisations could consider, particularly at a time when meeting the growing demand on mental health services may compromise the level of commitment to professional well-being.

### Limitations and Directions for Future Research

A previous review of quantitative studies on VPTG presented literature characterised by small sample sizes and a lack of rich comparison between professionals (Manning-Jones et al., 2015), both of which have shown great improvement in this review. The identified quantitative studies had a high number of participants, and growing studies have focused on comparisons between types of professionals in different settings. Nevertheless, the scope of the literature remains narrow. To our knowledge, this is the first review that used a mixed-design to provide an overview of the existing evidence base, including both quantitative and qualitative studies, on the topic of VPTG in professionals who work with trauma survivors. It is believed that this review incorporated research from around the world, and therefore can be considered culturally diverse.

A number of limitations were noted in relation to the existing literature. Firstly, there remains no validated measure to assess VPTG. All quantitative studies in this review used the Post-Traumatic Growth Inventory designed to measure growth in individuals who have experienced a traumatic event directly (Tedeschi & Calhoun, 1996) (rather than those affected vicariously, such as professionals). It has also been suggested that this measure has limited capacity to assess all aspects of VPTG, as does not allow for the different elements that are unique to vicarious rather than direct exposure to trauma to be considered appropriately (Manning-Jones et al., 2015). As such, an instrument measuring VPTG is overdue and necessary. Furthermore, this self-report measure, similar to other growth scales has been criticised for its items being positively biased (Ford et al., 2008), with participants naturally over-stating scores of growth items, as a result of how these are presented and desirability bias.

Finally, the literature today comprises close to no longitudinal studies on VPTG. All but one study included in the present review used a cross-sectional design, meaning that VPTG was measured at one specific point in time. There continues to be little to no understanding of the developmental process that underpins VPTG, which is considered to be a significant gap in the literature (Calhoun & Tedeschi, 2006; Joseph & Linley, 2008).

Future research is suggested to explore in more detail the personal, cognitive and behavioural aspects of professionals that can act as protective factors in their work and facilitate growth. These may include attitudes towards and perceptions around trauma and growth, as well as behavioural characteristics that promote it. Furthermore, so far, it remains unexplored how trauma narratives and experiences of vicarious trauma and VPTG in professionals are shaped by their gender identity, culture, religion, ethnicity, socioeconomic status and other markers. As such, there continue to be questions around the impact of one’s individual diversity and social context on VPTG that have the potential to inform more individualistic and self-reflective approaches in supervision and training.

## Conclusion

According to psychological theory, all organisms are naturally motivated toward growth (Rogers, 1961). However, the majority of research has focused on the negative impact of working with trauma survivors. As such, it seems crucial that research on the positive effects, and what may facilitate growth, continues to develop. Ultimately, this review demonstrates the importance of supplying a well-trained, supervised and self-reflective body of professionals to meet the increasing needs of traumatised individuals.

## Summary of Critical Findings

**Table:** 

Critical Findings
- Moderate levels of vicarious traumatisation are the strongest predictor of VPTG, with high levels impeding growth. - Professionals identified growth in (i) the way they perceived themselves, (ii) their philosophy of life and (iii) their interpersonal relationships. - High quality organisational support and positive relationships with colleagues and supervisors are crucial in mitigating vicarious trauma and facilitating growth. - Active use of social support and self-care strategies were linked to significant VPTG in professionals.

## Summary of Implications for Practice, Policy and Research

**Table:** 

	Implications
*Practice*	- The availability of resources for professionals’ well-being is vital. Organisations should provide adequate regular supervision and peer support, as well as training that educates professionals about vicarious trauma and practices of self-care. - Supervision and training can be used to encourage professionals to reflect on the work’s personal impact, both positive and negative. - Professionals should be supported to take regular breaks to process traumatogenic material, while maintaining their work-life balance.
*Policy*	- Development of policies around safeguarding professionals and adopting trauma-informed care within services. - Recruitment procedures, specialist training and supervision should take into account factors that have been found to predict vicarious trauma and growth within the work environment.
*Research*	- A better understanding of professionals’ experiences of growth as a result of their work with survivors of trauma. - Identification of organisational and personal factors that facilitate and contribute to growth. - Identification of significant gaps in the literature, including a measure for Vicarious Post-Traumatic Growth, longitudinal research designs and questions around the effect of professionals’ social contexts and individual diversity.

## Diversity Statement

A total of 1597 participants took part in the 15 studies included in the present review. Of these, 14 studies reported the gender of the participants. 27% of the participants were male and 73% were female, suggesting an over-representation of female participants in this field. The age of the participants ranged from 18 to 73 across the 11 studies that reported age, covering a wide range. Participants were mental health professionals working in both the private and public sectors, and their years of experience ranged from one to 50 years (based on the available data in 10 studies). The studies were conducted in various different countries around the world, with participants reporting multiple nationalities. As such, the sample across studies included in the present review can be considered ethnically diverse, representing both Western and non-Western countries. Nevertheless, the powerful experience that is VPTG will undoubtedly vary across gender identities, ages, religions, cultures and other markers. This review magnified the importance of future research using a targeted approach to explore this phenomenon and its diversity across different participants.

## Supplemental Material

sj-pdf-1-tva-10.1177_15248380221082079 – Supplemental Material for Vicarious Post-traumatic Growth in Professionals Exposed to Traumatogenic Material: A Systematic Literature Review Click here for additional data file. Supplemental Material, sj-pdf-1-tva-10.1177_15248380221082079 for Vicarious Post-traumatic Growth in Professionals Exposed to Traumatogenic Material: A Systematic Literature Review by Alexandra Tsirimokou, Juliane A. Kloess and Sonia K. Dhinse in Trauma, Violence, & Abuse
