# Supplementary material for: Vicarious Post-traumatic Growth in Professionals Exposed to Traumatogenic Material: A Systematic Literature Review
Source: Trauma Violence Abuse. 2022 Apr 29;24(3):1848–66. doi: 10.1177/15248380221082079 (PMC10240641; doi:10.1177/15248380221082079)
Supplement: sj-pdf-1-tva-10.1177_15248380221082079 – Supplemental Material for Vicarious Post-traumatic Growth in Professionals Exposed to Traumatogenic Material: A Systematic Literature Review [file sj-pdf-1-tva-10.1177_15248380221082079.pdf]

Quality table of the eight qualitative studies

**Study**		Barrington et al. (2014)	Coleman et al. (2021)	Hernandez et al. (2015)	Hyatt-Burkhard (2014)	Puvimanasinghe et al. (2015)	Ren et al. (2018)	Silveira & Boyer (2015)	Splevins et al. (2010)
Theoretical Approach	1. Is a qualitative approach appropriate?	Yes	Yes	Yes	Yes	Yes	Yes	Yes	Yes
	2. Is the study clear in what it seeks to do?	Yes	Yes	Yes	Yes	Yes	Yes	Yes	Yes
Study Design	3. How defensible /rigorous is the research design /methodology?	Defensible	Defensible	Partial	Defensible	Partial	Defensible	Defensible	Defensible
Data Collection	4. How well was the data collection carried out?	Appropriate	Appropriate	Partial	Appropriate	Appropriate	Partial	Appropriate	Appropriate
Trustworthiness	5. Is the role of the researcher clearly described?	Yes	Yes	Not described	Not described	Not described	Not described	Yes	Yes
	6. Is the context clearly described?	Yes	Yes	Yes	Yes	Yes	Unclear	Yes	Yes
	7. Were the	Yes	Yes	Partial	Yes	Partial	Yes	Yes	Yes

	methods reliable?								
Analysis	8. Is the data analysis sufficiently described*?	Yes	Yes	Partial	Partial	Yes	Yes	Yes	Yes
	9. Are the data ‘rich’?	Yes	Partial	Partial	Partial	Yes	Yes	Yes	Yes
	10. Is the analysis reliable?	Yes	Yes	Partial	Not reported	Not reported	Partial	Yes	Yes
	11. Are the findings convincing?	Yes	Partial	Partial	Partial	Yes	Yes	Yes	Yes
	12. Are the findings relevant to the aims of the study?	Yes	Partial	Partial	Yes	Partial	Yes	Yes	Yes
Conclusions	13. Are the contributions made by findings clearly described and grounded in previous literature?	Yes	Yes	Inadequate	Yes	Yes	Yes	Yes	Yes
	14. Are the strengths and limitations of the study identified and	Yes	Partial	Partial	Inadequate	Yes	Inadequate	Partial	Yes

	discussed?								
Ethics	15. How clear and coherent is the reporting of ethics?	Appropriate	Partial	Inadequate	Inadequate	Appropriate	Appropriate	Appropriate	Partial
	16. Were funding sources and conflicts of interest acknowledged?	Yes	Yes	Yes	Not addressed	Yes	Yes	Yes	Yes
Overall Assessment	(++, +, -)	++	+	-	+	+	+	++	++

Quality table of the seven quantitative studies

**Study**		Ben-Porat (2015)	Brockhouse et al. (2011)	Manning-Jones et al. (2016)	O'Sullivan et al. (2011)	Rhee et al. (2013)	Rizkalla et al. (2020)	Zerach et al. (2015)
Introduction	1. Were the aims/objectives of the study clear?	Yes	Yes	Yes	Yes	Yes	Yes	Partial

Methods	2. Was the study design appropriate for the stated aim(s)?	Yes	Yes	Yes	Yes	Yes	Yes.	Yes
	3. Was the sample size justified?	Yes	Yes	No	Yes	Yes	Yes	No
	4. Was the target/reference population clearly defined?	Partial	Yes	Yes	Yes	Yes	Yes	Yes
	5. Was the sample frame taken from an appropriate population base so that it closely represented the target/reference population under investigation?	Yes	Yes	Yes	Partial	Yes	Yes	Yes
	6. Was the selection process likely to select subjects/participants that were representative of the target/reference population under investigation?	Partial	Yes	Partial	Yes	Yes	Yes	Yes
	7. Were measures undertaken to address and categorise non-respondents?	No	Yes	No	No	No	No	No
	8. Were the predictor and outcome variables measured appropriate to the aims of the study?	Yes	Yes	Yes	Yes	Yes	Yes	Yes
	9. Were the predictor and outcome variables measured correctly using instruments/ measurements that had been trialled, piloted and published previously?	Yes	Yes	Yes	Yes	Yes	Partial	Yes
	10. Is it clear what was used to determine statistical significance and/or precision estimates?	Yes	Yes	Yes	Yes	Yes	Yes	No

	11. Were the methods (including statistical methods) sufficiently described to enable them to be repeated?	Partial	Yes	Partial	Yes	Partial	Yes	Partial
Results	12. Were the basic data adequately described?	Yes	Yes	Yes	Yes	Yes	Yes	Yes
	13. Does the response rate raise concerns about non-response bias? *(reversed item)	Unclear	Yes	Unknown	Unclear	No	No	Unknown
	14. If appropriate, was information about non-responders described?	Partial	Yes	No	Partial	No	Yes	Partial
	15. Were the results internally consistent?	Yes	Yes	Yes	Yes	Partial	Yes	Yes
Discussion	16. Were the results presented for all the analyses described in the methods?	Yes	Yes	Yes	Yes	Yes	Yes	Yes
	17. Were the authors' discussions and conclusions justified by the results?	Yes	Yes	Yes	Yes	Yes	Yes	Yes
	18. Were the limitations of the study discussed?	Yes	Yes	Yes	Yes	Yes	Yes	Yes
Other	19. Were there any funding sources or conflicts of interest that may affect the authors' interpretation of the results? *(reversed item)	Unknown	Unknown	Unknown	Partial	No	No	Unknown
	20. Was ethical approval or consent of participants attained?	Yes	Yes	Yes	Yes	Unknown	Yes	Yes
